# Safety and Performance of Postmarketing Breast Implants: An Integrated Review with Technovigilance Data

**DOI:** 10.3390/jcm14124164

**Published:** 2025-06-12

**Authors:** Antonio de Aracoeli Lopes Ramalho, Albaniza Alves Tavares, Henrique Nunes da Silva, Rômulo Feitosa Navarro, Victhor Alexandre Vilarins Cardoso da Silva, Stela Candioto Melchior, Maria Glória Vicente, Marcus Vinícius Lia Fook, Suédina Maria de Lima Silva

**Affiliations:** 1 Graduate Program in Materials Science and Engineering, Northeast Biomaterials Evaluation and Development Laboratory (CERTBIO), Academic Unit of Materials Engineering, Federal University of Campina Grande, Campina Grande 58429-900, PB, Brazil; aracoeli@uol.com.br (A.d.A.L.R.); henrique.nunes@certbio.ufcg.edu.br (H.N.d.S.); romulonavarro13@gmail.com (R.F.N.); marcus.liafook@certbio.ufcg.edu.br (M.V.L.F.); 2 National Health Surveillance Agency, General Management of Monitoring of Products Subject to Health Surveillance, Technovigilance Management, Brasília 71205-050, DF, Brazil; victhor.silva@anvisa.gov.br (V.A.V.C.d.S.); stela.melchior@anvisa.gov.br (S.C.M.); maria.vicente@anvisa.gov.br (M.G.V.)

**Keywords:** medical device safety, health surveillance, breasts, breast implants

## Abstract

**Background/Objectives**: Breast implants are widely used in reconstructive surgeries, as well as in cosmetic procedures, to enhance or restore breast shape and volume. With advances in techniques and materials, these devices have become safer and more effective over the years. Nevertheless, complications such as capsular contracture, rupture, infections, or other types of malignancies (BIA-SCC). This study evaluated the postmarketing safety and performance of implants via technovigilance data and a review of scientific studies. **Methods**: The research analyzed publications from the BVS, PubMed, Embase, and ClinicalTrials databases from between 2007 and 2023 (15 years), in addition to reports registered in the Notivisa system during the same period. **Results**: A total of 113 studies were identified, 15 of which were selected for the final analysis, which revealed that capsular contracture, seroma, infection, and rupture were the most common complications. In the Notivisa system, 786 reports were found, including 397 technical complaints and 389 adverse events, with pain, infections, and lymphoma among the most frequently reported issues. **Conclusions**: These findings highlight the importance of continuous surveillance to identify risks and promote improvements in the quality and safety of breast implants, ensuring patient well-being. As a practical contribution, a clinical decision-making algorithm was proposed to support healthcare professionals in the early identification and management of implant-related complications.

## 1. Introduction

Breast surgery with implants stands out as one of the most commonly performed procedures in plastic surgery, encompassing both aesthetic purposes, such as breast augmentation, and reconstructive purposes, such as partial or total mastectomy [1,2]. The available implants vary in filling type—saline solution or silicone gel, with different degrees of cohesiveness—and in surface characteristics, as they can be smooth, textured, or polyurethane-coated [3,4].

These devices are regulated by a set of standards that ensure their safety and biological compatibility. In Brazil, the resolutions of the National Health Surveillance Agency (ANVISA) establish specific guidelines, such as Resolution RDC No. 665/2022, which sets quality requirements for the production and commercialization of medical devices [5], and RDC No. 550/2021, which outlines the minimum requirements related to the identity and quality of breast implants, as well as the requirement for conformity certification [6].

Additionally, international standards, such as those of the International Organization for Standardization (ISO), play crucial roles in ensuring the safety and performance of breast implants. ISO 14607 Nonactive surgical implants—Mammary implants—Particular requirements (ISO, 2018) establishes safety and performance requirements for breast implants, whereas the NBR ISO 10993 standard provides guidelines for the biological evaluation of medical device materials [3,7]. ASTM F703-18 addresses requirements for silicone gel-filled and saline-inflatable breast prostheses [8], and ASTM F2051-00 outlines specifications for smooth, textured, inflatable, and single-use saline or silicone breast implants [9], both of which are intended for surgical reconstruction, augmentation, or replacement. The Guidance on Saline, Silicone Gel, and Alternative Breast Implants, published by the U.S. Food and Drug Administration (FDA), defines specific requirements for the American market, including material safety and integrity assessment methods [10].

The convergence of these national and international standards is essential for ensuring the safety and performance of breast implants in a global context, especially given concerns related to complications and risks associated with these devices.

However, the history of breast implants has not been controversial. In the 1990s, the FDA imposed a moratorium on silicone implants due to a lack of scientific evidence regarding their safety, a decision that led to their removal from the U.S. market until 2006, when further research allowed their reintroduction [11]. In 2010, new challenges emerged with the withdrawal of Poly Implant Prothèse (PIP) implants from the European market after the detection of improperly used industrial silicone instead of medical-grade silicone [12].

These controversies prompted improvements in the design and safety of breast implants. Since the first silicone models were developed by Cronin and Gerow in 1962, the design and materials of implants have evolved significantly. Early devices, with thick shells and dense gel, had an unnatural appearance and were associated with high rates of capsular contracture [4,13]. Second-generation implants feature thinner shells and less viscous silicone gel but face issues with gel diffusion [4]. Subsequent generations introduced silicone elastomer layers and more cohesive gels, enhancing the appearance and reducing complications such as capsular contracture and ruptures [13,14,15].

Despite advancements, complications associated with the use of breast implants, particularly implant rupture and capsular contracture, remain a concern. Rupture, which can occur due to trauma or wear, leads to rapid deflation in saline implants and insidious exposure of the gel in silicone implants [16,17]. Capsular contracture, characterized by the hardening of tissue around the implant, results in discomfort and aesthetic alterations [18]. Other complications include implant migration, seroma, and infection, with the latter being the most significant, as it compromises both patient health and device integrity [19,20,21]. Therefore, this integrated review aimed to evaluate the postmarketing safety and performance of breast implants, focusing on identifying major complications and analyzing data from technovigilance.

## 2. Methodology

The methodology of this study is descriptive, cross-sectional, and exploratory, employing both quantitative and qualitative approaches, and is divided into two stages. In the first stage, a review of the scientific literature was conducted to investigate the nature of issues related to the safety and performance of breast implants in the postmarketing phase. Databases such as the Regional Health Portal (BVS), PubMed, Embase, and the clinical trial registry ClinicalTrials were consulted, using publications from the period between 2007 and 2023. This timeframe corresponds to the data collection period for reports registered in the National Health Surveillance Notification System (Notivisa).

The search terms included “Breast Implantation,” “Breast Implants,” “Safety,” “Performance,” “Product Surveillance,” “Postmarketing,” “Silicone Gel Implant,” “Polyurethane Implants,” “Adverse Events,” “Technical Complaints,” “Smooth Surface Implants,” and “Textured Surface Implants,” along with their synonyms, combined with Boolean operators (AND and OR). The proposed research question aimed to investigate the most common complications and the key factors affecting the safety and performance of breast implants in the postmarketing phase.

Studies that evaluated the safety and performance of breast implants in the postmarketing phase were included. Observational studies, including prospective and retrospective cohort studies, descriptive and multicenter studies, and experimental and clinical studies, were considered. The selection included studies published in English, Portuguese, French, or Spanish between 2007 and 2023. Eligible studies were those that addressed the complications, adverse effects, and functional efficacy of breast implants, covering different types of surfaces (smooth or textured) and materials (silicone, polyurethane, or saline solution).

Studies that did not provide specific information on the safety and performance of implants, those whose focus was another type of medical device, and publications belonging to other study designs, such as case reports, books, and book chapters, were excluded. Systematic reviews were excluded if they did not present relevant primary data for the analysis. Additionally, articles funded by companies, studies based solely on the experience of a single physician, or research that only assessed patient satisfaction were not considered.

To assess the methodological robustness of the included studies, a hierarchical system for classifying levels of evidence was adopted, ensuring greater rigor in the interpretation of the findings. The classification followed a model that categorizes studies as follows: (i) Level 1—systematic reviews or meta-analyses of randomized controlled clinical trials; (ii) Level 2—well-designed randomized controlled clinical trials; (iii) Level 3—well-designed clinical trials but without randomization; (iv) Level 4—well-designed cohort and case-control studies; (v) Level 5—systematic reviews of descriptive and qualitative studies; (vi) Level 6—individual descriptive or qualitative studies; (vii) Level 7—expert opinions and committee reports [22]. The classification of the studies was carried out independently by two researchers, with any disagreements resolved by consensus.

In the second stage, real-world data analysis was conducted using technovigilance notifications related to breast implants registered in the Notivisa system between 1 January 2007 and 31 December 2023. Data extraction was performed in October 2024, with 786 notifications for products registered under the technical names “Breast Prostheses” (108 adverse events and 63 technical complaints) and “Breast Implants” (281 adverse events and 334 technical complaints). Importantly, the Notivisa system is dynamic, which may result in discrepancies in the numbers presented in future queries. The real-world database, provided anonymously by the Technovigilance Management (Getec) of ANVISA, contains reported information on technical complaints and adverse events. The notifications were categorized by the type of occurrence, year, and type of adverse event, allowing for the quantification of occurrences. These data were electronically organized and presented in graphs and tables to facilitate the analysis of the observed patterns.

## 3. Results and Discussion

### 3.1. Study Selection

A bibliographic search was conducted in December 2024 via the search sequence described in the Supplementary Material (Table S1). The sensitivity and refinement applied in the search strategy resulted in a total of 113 publications retrieved from the following consulted databases: Regional Health Portal—BVS, PubMed, Embase, and ClinicalTrials.

**Figure 1:**
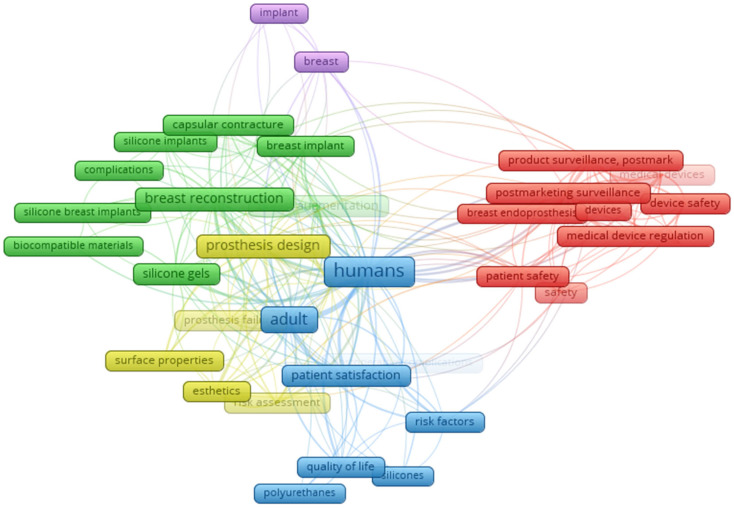
Co-occurrence and connectivity of terms found in titles, keywords, and abstracts of publications retrieved from the databases. Analysis was performed via VOSviewer 1.6.20 software (2023), with a minimum of 2 occurrences and a “total count” setting. Sources: BVS, PubMed, Embase and ClinicalTrials (2024).

The bibliometric network of recurring terms found in the titles, keywords, and abstracts of these documents, which appeared at least 22 times, was configured with “co-occurrence,” “full count,” and “keywords,” as illustrated in Figure 1. The analysis provides an overview of the most frequent terms, highlighted by larger rectangles. The academic community’s focus encompasses topics such as humans, implants, breasts, biocompatible materials, capsular contracture, prosthesis design, silicone gels, surface properties, aesthetics, patient satisfaction, quality of life, breast implants, postmarketing product surveillance, safety, postmarketing surveillance, silicone, polyurethane, and risk factors, among others.

From the initial identification of 113 relevant studies on the safety and performance of breast implants, 5 (4.42%) were excluded because of duplication, resulting in 108 (95.58%) for title and abstract screening. From this screening, 52 studies (48.15%) were eliminated, leaving 56 (51.85%) publications for full-text review. During this stage, 41 articles (73.21%) were excluded because they did not meet the eligibility criteria listed in Section 2. This methodology resulted in a final analysis of 15 studies (26.79%), of which 7 (46.67%) were clinical trial protocols (CTs) and 8 (53.33%) were scientific articles.

The scientific articles cover research conducted in various countries, including the Netherlands [23,24], the United Kingdom [25], Germany [26,27], Italy [28], the United States [29], and South Korea [30]. The clinical trials (CTs) that include postmarketing investigations and prospective trials are from the United States [31], Brazil [32,33], and France [34,35], and the global multicenter CTs involve brands such as Motiva^®^ [36] and Nagor PERLE [37].

The annual distribution of the publications demonstrates a growing interest in research on breast implants. In terms of clinical trials, publications occurred in 2012 (one study), 2018 (two studies), 2022 (two studies), and 2023 (two studies). The scientific articles were published in 2013 (two articles), 2015 (two articles), 2016 (one article), 2018 (one article), 2021 (one article), and 2022 (one article). This pattern indicates increasing attention to the safety and performance of breast implants over the past decade, with a notable rise in clinical study registrations, reflecting the growing interest in postmarketing evaluation of these devices.

This variation in the volume of publications reflects interest in discussions about the safety of breast implants, which is closely linked to regulatory changes and advances in surgical techniques over the years. The characteristics of the studies included in this review provide detailed information on the safety and performance of breast implants, identifying the main complications associated with these devices and contributing to a broader understanding of the risks involved.

### 3.2. Classification of the Level of Evidence of the Included Studies

The classification of the levels of evidence for the studies included in this review (Table 1) followed a hierarchical system that considers methodological robustness and the degree of reliability of the findings. The analyzed studies vary in design, ranging from prospective and retrospective cohorts to experimental and descriptive studies. Prospective and retrospective cohort studies represent most of the sample and are classified as Level 4. Examples of studies in this category include those by Maijers et al. [23], Brunnert [26], De Lorenzi et al. [28], Hong et al. [30], and Miseré and Van Der Hulst [24]. These studies are considered relevant sources of evidence, as they analyze patient groups over time, allowing for the observation of associations between exposure factors and clinical outcomes. However, the absence of randomization reduces the strength of causal inference.

The study by Yildirimer et al. [25], an experimental study, was classified as Level 6. This level of evidence generally includes laboratory or preclinical studies, and it contribute significantly to knowledge but has limitations in direct extrapolation to clinical practice.

Retrospective and descriptive studies, such as those by Hammond and Schmitt [29] and Huemer et al. [27], were also classified as Level 6. These studies provide important information on clinical patterns and adverse events; however, since they do not establish structured comparisons between groups, they have a lower level of evidence.

The predominance of Level 4 studies indicates that most of the available evidence derives from well-designed observational cohorts, which, although valuable, are limited in establishing causal relationships due to the absence of randomized controlled trials (Levels 1 and 2). The inclusion of Level 6 studies, with descriptive and exploratory approaches, adds breadth to the analysis but requires cautious interpretation regarding clinical applicability. This distribution reflects not a lack of scientific value, but the inherent methodological constraints of research on implantable medical devices, where ethical considerations, long-term follow-up requirements, and intervention standardization pose significant challenges to conducting RCTs.

Nonetheless, the incorporation of observational studies is consistent with international methodological standards. Organizations such as the National Institute for Clinical Excellence (NICE) and the Food and Drug Administration (FDA) recognize the importance of real-world data and observational evidence in postmarketing surveillance and regulatory decision-making. Therefore, while the current evidence base is limited in methodological rigor, it remains highly relevant and aligned with contemporary practices in technovigilance and clinical safety monitoring.

### 3.3. Characteristics of the Included Articles (n = 8)

The studies analyzed in this article investigated the safety and performance of breast implants in the postmarketing phase, with a focus on the main identified complications. Table 2 provides an overview of the descriptive characteristics of the studies included in the review.

The safety and performance of breast implants remain central topics in the medical literature. This review identified and analyzed the main risks associated with the use of these devices, correlating these complications with real-world data, particularly technical complaints and adverse events reported in the Notivisa system during the postmarketing phase. The complications associated with breast implants, as reported in the included studies, highlight a variety of issues that can impact the safety and performance of these devices. The most frequently cited complication was capsular contracture, reported in seven studies [23,24,25,27,28,29,30]. This condition is characterized by hardening of the tissue around the implant, which can cause pain, discomfort, and aesthetic deformity. In many cases, capsular contracture requires reoperation to relieve symptoms and restore the implant’s appearance [18].

Seroma, identified in three studies, is another serious complication [26,29,30]. It manifests as fluid accumulation around the implant, usually in the postoperative period. This accumulation can often be resolved through drainage, but in more severe cases, there is a risk of infection or other complications [19]. Infection, reported in five studies [23,24,28,29,30], is another significant concern and can arise from factors related to surgery or implant handling. Severe infections often require implant removal for effective treatment [38]. Implant rupture was reported in four studies and is considered a relevant complication [24,25,27,28]. Rupture occurs when the integrity of a material is compromised, due either to natural wear or trauma, and generally necessitates implant replacement [1]. Another common issue is hematoma, which was cited in two studies [29,30]. This accumulation of blood around the implant requires intervention to prevent additional complications, such as infection and capsular contracture [39]. However, bruising appears shortly after surgery and may also be related to the surgical technique.

Implant malposition was reported in two studies [27,28] and can lead to asymmetry [24,28,30], sometimes requiring additional surgery to correct the position of the implant. Persistent pain, mentioned in three publications [23,24,25], is a significant complaint that may be linked to various causes, such as infection, capsular contracture, or nerve injury. Other less frequent complications, such as breast cancer, tissue necrosis, and aesthetic changes, were also reported in the studies included in this review. Although these occurrences are less common, they can still have a substantial impact on patients’ health and quality of life. In many cases, these complications require additional interventions, such as further surgeries or rehabilitation treatments.

Maijers et al. [23] analyzed 80 women with silicone breast implants, with a mean age of 47 years, who reported unexplained systemic symptoms, most of which were related to aesthetic surgery. The main symptoms observed—commonly associated with Breast Implant Illness (BII)—were fatigue (89%), joint pain (69%), and muscle pain (65%). Additionally, 75% of the participants had preexisting allergies, and all met at least two criteria for autoimmune/inflammatory syndrome induced by adjuvants (ASIA). Among the 52 women who underwent explantation, 36 reported a significant reduction in symptoms, while 9 reported complete resolution. Although the study suggests a possible association between silicone implants and systemic symptoms, particularly in patients with a history of allergies, it is important to consider potential biases in the analysis of the results. It is noteworthy that ASIA syndrome is not definitively classified under Breast Implant Illness (BII), although it is often discussed within this context. In contrast, the term “Breast Implant-Associated” (BIA) typically refers to malignant conditions such as BIA-ALCL and BIA-SCC. Furthermore, BII symptoms may persist in approximately 25–30% of cases even after explantation. Selection bias may have occurred due to the recruitment of participants through national media, which may have attracted women with negative experiences or those predisposed to link their symptoms to silicone implants, resulting in a nonrepresentative sample. Confirmation bias could also have influenced the physicians, who, when examining patients already convinced of the relationship between their symptoms and the implants, might have interpreted the signs in a biased manner. Additionally, recall bias could have affected the accuracy of reports on the onset and progression of symptoms, as the participants may have been influenced by their own experiences and the reports of others.

Yildirimer et al. [25] analyzed the mechanical and surface properties of Poly Implant Prothèse (PIP) silicone breast implants that were explanted due to complications arising from the use of nonmedical-grade silicone gel in their manufacturing process. They demonstrated that the capsules of these implants had significantly lower resistance than did medical-grade silicone implants. Additionally, a negative correlation was observed between the mechanical properties and implantation time, indicating progressive material degradation in vivo. ATR-FTIR analyses revealed structural changes associated with the breakdown of chemical bonds over time. The implants were removed due to various complications, including capsular contracture, implant rupture, severe pain, bilateral sensitivity, displacement, and nipple discharge. In some cases, explantation was performed prophylactically due to breast discomfort or as part of reconstructive and aesthetic correction procedures. The Poly Implant Prothèse, which had a global distribution, was recalled, and thousands of users underwent surgery for implant removal.

Brunnert [26] evaluated Diagon/Gel^®^ 4Two implants coated with micropolyurethane foam in 90 patients who underwent aesthetic and reconstructive breast surgeries, resulting in a total of 152 implants. The study, which was conducted in a single center by one surgeon, included both primary and reoperative procedures, with most indications focused on oncological reoperation (34.2%) and aesthetic procedures (28.3%). With an average follow-up time of 41 months, the preliminary results indicated a very low complication rate, with no cases of capsular fibrosis, rotation, or implant rupture. However, the absence of a control group and the relatively short follow-up period emphasize the need for additional studies to validate these findings in the long term.

De Lorenzi et al. [28] investigated 578 patients with 658 Poly Implant Prothèse anatomical implants, with an average volume of 280 cc and an average age of 50 years. Among these, 443 implants (67.3%) were explanted, with 409 performed at a single institute and 34 at other centers. The main reasons for implant removal included asymmetry, capsular contracture, and rupture. Before the safety alert in 2010, the rupture rate was 11.6%, whereas after the alert, this rate increased to 24.7%, with an average lifespan of 91 months in the second group. Although most of the explanted implants were intact, 5.4% showed silicone leakage. The study highlighted the importance of rigorous postoperative monitoring, as well as immediate measures to prevent complications, emphasizing concerns about the safety and effectiveness of Poly Implant Prothèse implants.

The study by Hammond and Schmitt [29] on the McGhan Style 153 double lumen breast implant highlighted a high rate of long-term complications, with a rupture rate of 36.6% of implants and significant capsular contracture in 51.5% of cases. Ruptures were more frequent in the upper pole of the implants, and the detection of failures predominantly occurred through clinical examination. The implant removal rate was 77.6%, with an average time of 7.5 years until removal. Failures were associated with the double-lumen implant design, which created stress points and failures due to wrinkling of the shell. These results underscore the importance of improving shell stability and modifying the design of implants to minimize these issues in future generations of breast implants. However, some biases should be considered when interpreting the results of this study. Selection bias may have occurred because the sample consisted of patients followed in a specific center, which may not reflect the general experience with the implant. Additionally, recall bias may have influenced the perception of symptoms and the reporting of complications, as the patients were followed over an extended period, potentially distorting the timing of some complications. Confirmation bias may also have influenced the results, as the assessment of implant failure was performed by a team of professionals who, based on the patients’ history, might have reinforced the association between the implant design and the observed complications. These limitations should be considered when conclusions about the safety and efficacy of McGhan Style 153 implants are analyzed.

The study by Huemer et al. [27] analyzed 100 primary breast augmentations performed with Motiva Ergonomix Round SilkSurface silicone implants over three years, focusing on safety and postoperative outcomes. Most of the patients had hypoplasia (52%) and ptosis (28%), with an average implant volume of 370 cc. The surgical approach used was an inframammary, with submuscular placement. The complication rate was 7%, including implant displacement, one rupture, and the need for aesthetic exchange in one patient. Despite the observed complications, the study highlighted a high satisfaction rate among patients and surgeons. However, the study has potential biases, such as selection bias (the sample may not be representative of the general population of implant patients), follow-up bias (with a minimum follow-up period of six months, which may not be sufficient for detecting long-term complications), and reporting bias (with the possibility of more positive reports due to expectations or the desire for favorable results).

Hong et al. [30] conducted a multicenter, retrospective study to assess the safety of breast augmentation with the Motiva Ergonomix™ Round SilkSurface implant in 873 Korean women over 4 years. The study analyzed postoperative complications in 1746 breasts, with an overall complication rate of 12.70%, including early seroma (2.70%), hematoma (2.10%), and capsular contracture (2.10%). The estimated time for the occurrence of complications was 918.34 days. Importantly, the study was retrospective, which raises the possibility of recall bias, as complications were based on patients’ memories or available records, which may not be complete.

Miseré and Van Der Hulst [24] reviewed breast implant explantation procedures performed between 2010 and 2020, with the aim of assessing the symptoms reported by patients preoperatively and the effects of explantation, particularly in women with suspected breast implant illness (BII). Although BII was the fifth most common cause of explantation, accounting for 11.2% of cases, the prevalence of this condition was considered relatively low. Approximately 60% of the patients diagnosed with BII reported significant improvement in symptoms after implant removal, with a particularly notable benefit among those who underwent autologous breast reconstruction. Additionally, the study revealed that a history of allergies and implant ruptures increased the likelihood of developing BII, with odds ratios of 2.1 for both factors. However, it is important to consider some potential biases that may have influenced the study’s results. The absence of a control group and the lack of randomization limit the generalizability of the findings, as there was no comparison between the patients who underwent explantation and those who kept their implants or did not develop BII symptoms. The reliance on medical records and subjective reports may introduce assessment bias, as there is no guarantee that all symptoms and health conditions associated with BII are fully documented. Finally, the lack of a prospective, long-term follow-up weakens the ability to assess the efficacy of explantation as a treatment for BII. The results suggest that while BII is a concern for some women, it does not represent a primary indication for explantation in most cases. This research emphasizes the need for prospective, controlled studies to confirm the benefits of explantation as an effective therapy for BII, particularly with respect to improvements in patients’ quality of life. The ratio of cure/improvement/no response after explantation in BII remains a topic of debate and requires future research. Additionally, the results suggest that autologous breast reconstruction may be a valuable option for those seeking effective treatment after explantation.

The analysis of the articles reveals that although breast implants offer significant benefits in terms of reconstruction and aesthetics, the available data reinforce the importance of constant monitoring to identify and minimize the risks associated with these devices. While current standards and protocols provide a high level of safety, it is necessary to continuously improve postmarket surveillance and strengthen regulatory systems to ensure a swift and effective response to patient safety concerns.

### 3.4. Characteristics of the Included Clinical Trials (n = 7)

The safety and performance of breast implants are important issues to be evaluated in aesthetic and reconstructive medicine. The clinical trials included in this study aimed to investigate the safety and performance of breast implants in the postmarket phase. The results obtained from these clinical trials can provide a comprehensive assessment of the safety and effectiveness of various brands and types of silicone implants using different monitoring approaches and outcome metrics. They focus on short- and long-term safety and patient satisfaction and analyze the frequency and severity of adverse events and complications associated with the implants.

Clinical trials are essential for generating robust evidence on the safety and effectiveness of medical devices, allowing for a thorough analysis of the associated risks and benefits. In the context of breast implants, these studies provide more accurate information that can assist both healthcare professionals in making clinical decisions and patients in accessing reliable data to choose the most suitable implant type for their needs, as well as the manufacturer. The evidence from clinical trials helps improve the product while minimizing the risks associated with its use.

With respect to long-term safety and adverse events, studies such as the Sientra study (ClinicalTrials.gov Identifier: NCT01639053) and those on Silimed^®^ implants (ClinicalTrials.gov Identifiers: NCT03356132 and NCT05345821) focus on analyzing adverse events; conducting periodic assessments every three years; and considering variables such as the incidence of infections, capsular contracture, and ruptures. This approach allows for detailed observation of complications that may arise over time, providing robust insight into the safety of implants during prolonged use.

After the approval of Sientra silicone gel breast implants in March 2012, the U.S. Food and Drug Administration required a 10-year postapproval study (ClinicalTrials.gov: NCT01639053). The authors presented the results of the first six years of this study, which involved 5197 patients (10,327 implants) and focused on the clinical performance of the implants in breast augmentation, reconstruction, and revision procedures. The analyses revealed a 4.1% risk of grade III/IV capsular contracture, an 11.6% reoperation rate, and a 7.8% implant removal rate. Over 50% of the reoperations were aesthetic. The data were consistent with those of the main study, and there were no cases of anaplastic large-cell lymphoma associated with the implants [40]. Table 3 provides a summary of the clinical trials included in this study.

In addition to safety, some protocols include the assessment of patient and surgeon satisfaction via questionnaires such as the BEQ-Brazil and the Rosenberg Self-Esteem Scale. These measures are essential for understanding the impact of implants on aesthetic perception and quality of life, providing subjective data that complement safety analyses. For example, in the Motiva^®^ Sizer clinical trial (ClinicalTrials.gov Identifier: NCT06274736), groups will be divided to assess the incidence of complications and satisfaction of both patients and surgeons, allowing for an evaluation of perspectives on the impact of using sizers (temporary devices to determine the desired size before surgery) on the need for reoperation or overall satisfaction with the results.

The protocols also focus on comparing different surfaces and types of implants. The analysis involving smooth, textured, microtextured, and polyurethane-coated implants, such as those performed with Silimed^®^ implants (ClinicalTrials.gov Identifier NCT03356132 and ClinicalTrials.gov Identifier NCT05345821) and SYMATESE AESTHETICS ESTYME^®^ MATRIX (ClinicalTrials.gov Identifier NCT03386682 and ClinicalTrials.gov Identifier NCT05336526), is crucial to identify which textures offer the lowest incidence of complications, such as capsular contracture or seromas. Textured surfaces, for example, are explored to prevent the formation of firm capsules around the implants, influencing the choice of implant in specific breast augmentation or reconstruction situations.

The clinical trial using the PERLE breast implant line from Nagor (ClinicalTrials.gov Identifier: NCT06013514) also examined reoperation rates, local complications, and long-term satisfaction. The analysis of local complications and the need for reoperation is crucial for creating a detailed risk profile of the implants and identifying which additional interventions may be necessary to maintain the safety and aesthetics of the implants over the years.

Postapproval trials are important for technovigilance processes, both at the state level and for manufacturing companies, as they provide crucial data on the safety and performance of breast implants under real-world conditions. This information helps improve surgical practices, monitor safety, shape public health policies, and support advances in implant design and materials. Additionally, they allow for improvements in regulatory and normative processes based on clinical evidence.

### 3.5. Analysis of Real-World Breast Implant Usage Data

**Figure 2:**
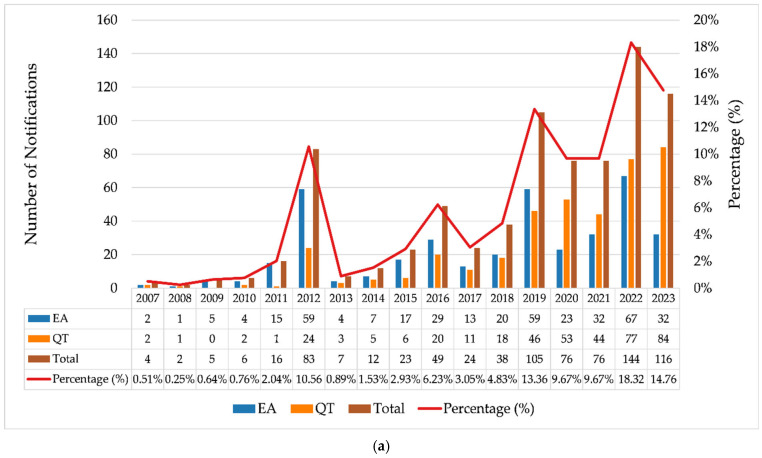

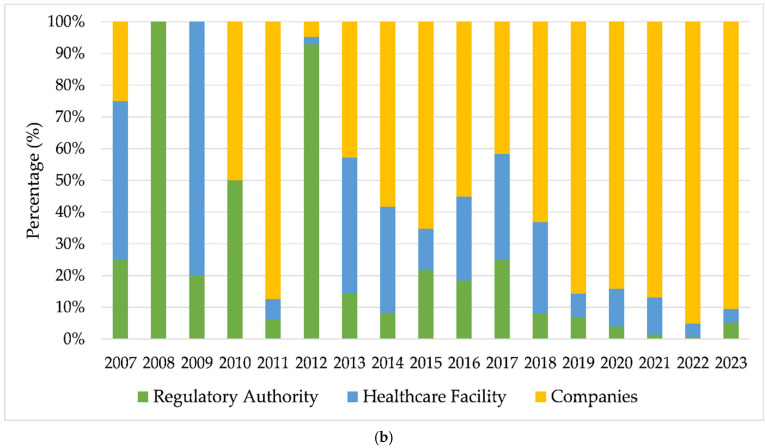
(**a**) Annual evolution of adverse event (AE) and technical complaint (TC) reports related to breast implants (2007–2023); (**b**) temporal evolution of the proportion of reports made by regulatory bodies, healthcare facilities, and companies. Source: ANVISA. Notivisa. The data were updated on 10/09/2024 and are subject to review.

Data on the real-world use of breast implants, collected through technical complaint (TC) and adverse event (AE) notifications registered in the Notivisa system, were analyzed for the period from 2007 to 2023. Figure 2a illustrates the evolution of AEs and TCs over this period, along with the relative percentage of occurrences throughout the years. Figure 2b presents the annual proportion of notifications segmented into three categories of reporters: regulatory authorities (ANVISA, State Visa, and Municipal Visa), healthcare establishments (hospitals, sentinel hospitals, and healthcare facilities), and companies.

In 2012, there was a substantial increase in notifications of AEs (*n* = 59) and TCs (*n* = 24) (Figure 2a), indicating a shift in behavior with intensified surveillance by regulatory authorities, which accounted for 92.8% of the notifications in that year (Figure 2b). This significant increase was likely driven by the scandal involving implants adulterated with industrial silicone by a French manufacturer and successive recalls of breast implants that occurred during that period, highlighting the importance of continuous surveillance in this context [41,42,43]. The publication of resolutions such as RDC No. 16 on 21 March 2012 [44] and RDC No. 33 on 3 May 2012 [45] significantly influenced the regulation of breast implants and prostheses in Brazil by establishing strict quality requirements, addressing previous regulatory gaps. Later, both resolutions were updated by RDC No. 550 on 22 December 2021 [6].

From 2013 onward, the proportion of notifications made by companies increased substantially, and this became responsible for most of the notifications in the following years. This was likely due to greater adherence to RDC Resolution No. 67, dated 21 December 2009, which made reporting mandatory for registration holders, as well as to Decree No. 8077 of 14 August 2013, which regulates the mandatory reporting of adverse events and technical complaints for all products under health surveillance [46].

For the following years, a growth trend was observed, although with fluctuations in the absolute number of notifications. Additional peaks are visible for 2016, 2019, and 2022, with 2022 showing the highest total volume of notifications (*n* = 144). Of these, 95.14% (*n* = 137) were made by companies, with 91 notifications made by a single registration holder in Brazil, consisting of 58 technical complaints (QT) and 33 adverse events (EA). A total of 20 of the EA notifications mentioned occurrences of BIA-ALCL (breast implant-associated anaplastic large cell lymphoma).

**Figure 3:**
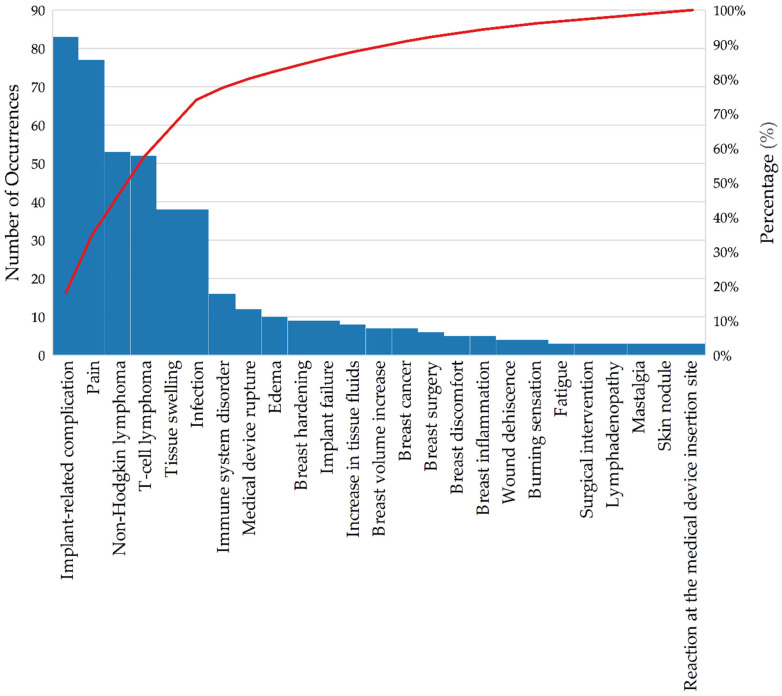
Pareto chart of the occurrence of adverse events associated with breast implants—Brazil, 2007–2023. Source: ANVISA. Notivisa. The data were updated on 10 September 2024 and are subject to review.

The data on adverse events (AEs) (Table 4 and Figure 3) related to breast implants recorded in the Notivisa system provide an important overview for understanding the health risks associated with these devices. Using the WHO-ART terminology from the World Health Organization, the AEs are classified and quantified in terms of relative and absolute frequency, allowing for the analysis of which problems occur most frequently.

The general term “implant-related complication” (13.3%) was the most common recurrent adverse event (AE), with 83 occurrences. These complications may include capsular contracture and misplacement, which are frequently reported in the literature, as noted in the studies included in the literature review [23,24,25,26,27,28,29,30]. Pain, with 77 occurrences (12.3%), is the second most common AE; it is also a frequent issue reported in the literature, and it is associated with various causes, such as infection, capsular contracture, and nerve injury. Although it is a less severe complication than other methods, its high frequency indicates a significant impact on patients’ quality of life. Non-Hodgkin lymphoma (8.5%) and T-cell lymphoma (8.3%) account for more than 16% of the adverse events reported in Notivisa and are also documented in the literature as conditions associated with breast implants. These data are particularly relevant, as they highlight the need for continuous monitoring of patients with breast implants to detect the emergence of these complications [41,47,48].

Infection and tissue swelling (6.1% each) are AEs that are frequently associated with postoperative complications, possibly related to inflammation or implant rejection. These findings are indicative of an adverse response of the body to the implant or problems during the recovery process. With 16 records (2.6%), immune system disorders highlight potential systemic reactions that may occur as a result of breast implants, suggesting an immune system response [49]. Several events with a frequency of less than 2%, such as product rupture (1.9%) and breast cancer (1.1%), were observed. Although their rates are lower, these AEs have a potentially serious impact and should not be overlooked.

In the “Other” category, AEs with only one or two occurrences were grouped to focus attention on the more frequent events and reduce the dispersion of results. The “not reported” category, which corresponds to AEs not specified at the time of the notification, represents an important aspect of the analysis. In the dataset extracted from the Notivisa system, these events total 72 occurrences, accounting for 11.5% of the total. The lack of detail in the records makes accurate identification of the type of AE that occurred difficult. This can compromise the effectiveness of preventive and corrective actions, as without the nature of the specified event, interventions are hindered [50].

The Pareto chart of adverse events (AEs) (Figure 3) related to breast implants provides a clear overview of which issues occur most frequently.

The analysis revealed that a small number of adverse events accounted for most of the adverse events. Approximately 80% of adverse events are caused by five main issues: implant-related complications (13.3%), pain (12.3%), non-Hodgkin lymphoma (8.5%), T-cell lymphoma (8.3%), and tissue swelling and infection (both 6.1%).

Real-world data highlight the main complications related to breast implants. The high occurrence of lymphomas associated with this type of material underscores the importance of ongoing medical monitoring for early detection of such conditions [41,51].

## 4. Clinical Decision-Making Algorithm and Recommendations for Postmarketing Safety Monitoring of Breast Implants

**Figure 4:**
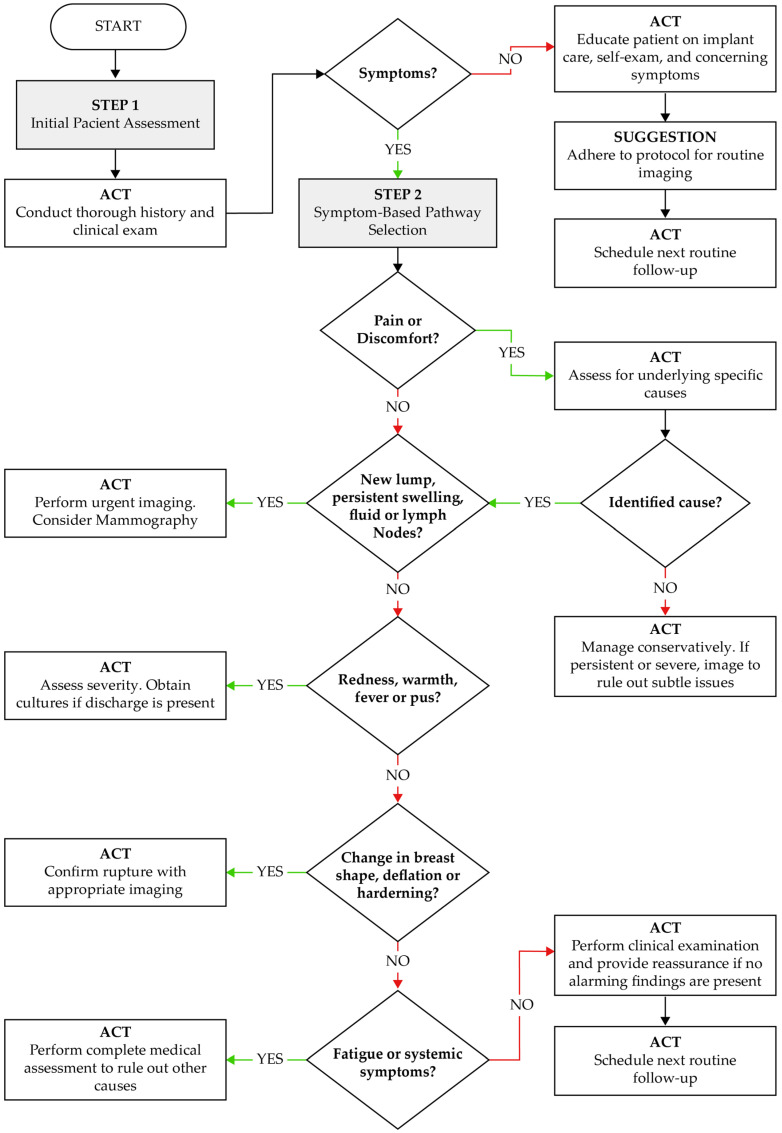
Clinical algorithm for the evaluation and management of patients with breast implants, based on symptomatology and postmarketing technovigilance data. ACT: Action to be taken by the healthcare provider based on the clinical scenario.

Based on the integrated analysis of technovigilance data and real-world clinical outcomes, we propose a symptom-based clinical decision-making algorithm to support early identification and appropriate management of complications related to breast implants (see Figure 4). This algorithm aims to aid clinicians in systematic evaluation and streamline decisions regarding imaging, patient reassurance, conservative treatment, or further investigation.

The algorithm begins with a structured patient assessment, emphasizing the need for a thorough clinical history and physical examination. Patients presenting with symptoms such as pain, lumps, swelling, fever, or changes in breast morphology are triaged through a stepwise process. Each step guides clinical action—ranging from urgent imaging and microbiological assessment to conservative follow-up—according to the nature and severity of findings. For asymptomatic patients, the algorithm recommends education on self-examination, adherence to routine imaging protocols, and follow-up scheduling.

This decision tool was designed in alignment with patterns identified in technovigilance reports, which highlight that delayed recognition of complications (e.g., seroma, capsular contracture, implant rupture, or infection) often leads to poor outcomes. By providing clear clinical triggers and actions, this model supports early detection and improves implant safety monitoring in routine practice.

## 5. Study Limitations

The limitations of this study include the heterogeneity of the data from the included studies, which made it difficult to compare results due to the diversity of protocols, types of implants evaluated, and data collection methodologies. The predominance of retrospective studies and the scarcity of high-quality randomized clinical trials (level 1 and 2) restrict the ability to establish robust causal conclusions. Furthermore, the lack of long-term follow-up in many of the evaluated studies prevents a comprehensive analysis of persistent complications, such as capsular contracture and infections, limiting the understanding of implant safety over time. These limitations highlight the need for more studies with standardized protocols, longer durations, and methodological rigor to strengthen the evidence on the safety and performance of breast implants.

Another limitation of this study is the absence of a direct comparative analysis between Brazilian postmarketing surveillance data (Notivisa) and international surveillance databases, such as the Manufacturer and User Facility Device Experience (MAUDE system FDA), European Database on Medical Devices (Eudamed), and Therapeutic Goods Administration (TGA). Such comparisons could offer valuable insights into global trends in adverse event reporting, enhance the contextual interpretation of safety profiles, and help identify regional discrepancies in device performance and reporting standards.

Another important limitation relates to the level of evidence of the included studies. Although this review adopted a structured evidence classification system, the majority of the studies were classified as Level 4 or 6, consisting mainly of observational cohorts and descriptive analyses. The scarcity of randomized controlled trials (RCTs) in this area reflects not only methodological gaps but also ethical and logistical challenges inherent in conducting RCTs with implanted medical devices, particularly in long-term surveillance contexts. Nonetheless, international guidelines such as those of the National Institute for Clinical Excellence (NICE) and the Food and Drug Administration (FDA) support the use of well-designed observational studies, particularly in postmarketing surveillance and technovigilance frameworks. These studies are considered valid sources of evidence for safety monitoring, risk signal detection, and regulatory decisions involving implantable medical devices. Therefore, although the evidence base is limited in rigor, it remains indispensable for regulatory insights and clinical vigilance.

Two main limitations were identified in the real-world data provided: (1) the presence of notifications with the generic term “Complications related to the implant” without detailing the specific nature of the complication observed. The lack of this specificity may hinder the identification of patterns and the adoption of more targeted measures to mitigate risks. (2) Notifications without proper completion of the adverse event coding field, recorded only as “Not informed,” compromising the categorization and accurate quantification of events, as well as making it difficult to assess the real impact of the reported adverse events. These limitations highlight the importance of improving the notification process and encouraging the provision of more detailed and standardized information.

## 6. Conclusions

The analysis of technovigilance data revealed a significant increase in notifications of adverse events and technical complaints related to breast implants, especially from 2012 onward. Complications such as pain, infection, tissue swelling, and lymphomas have emerged as the most common occurrences, highlighting the importance of continuous surveillance and improvements in the surgical care process.

The literature data corroborate real-world data regarding product behavior. It is important to emphasize the need for reporting problems encountered during the use of breast implants by regulatory authorities so that timely and comprehensive measures can be adopted regarding the safety and performance of these products.

The frequency of notifications with incomplete information points to the need for more detailed records to improve investigative actions triggered by notifications, as well as potential corrective and preventive measures. This review of the safety and performance of breast implants in the postmarket phase has expanded the understanding of the risks and benefits of these devices in a regulatory and technological advancement context.

To strengthen this surveillance system, we recommend implementing centralized digital implant registries, standardized electronic reporting tools within electronic health records, and accessible patient reporting platforms. Broader enforcement of unique device identification, the integration of clinical decision support tools, and the use of anonymized performance dashboards are also essential. These measures, combined with structured clinical algorithms, can significantly improve traceability, the quality of adverse event reporting, and the overall safety monitoring process.

## Figures and Tables

**Table 1:** Classification of the level of evidence of studies.

Author, Year	Study Design	Level of Evidence
Maijers et al., 2013 [23]	Descriptive cohort study	Level 4
Yildirimer et al., 2013 [25]	Experimental study	Level 6
Brunnert, 2015 [26]	Prospective cohort study	Level 4
De Lorenzi et al., 2015 [28]	Prospective cohort study	Level 4
Hammond and Schmitt, 2016 [29]	Retrospective and descriptive study	Level 6
Huemer et al., 2018 [27]	Retrospective study	Level 6
Hong et al., 2021 [30]	Multicenter and retrospective study	Level 4
Miseré and Van Der Hulst, 2022 [24]	Retrospective study	Level 4

**Table 2:** Overview of the descriptive characteristics of the studies included in the review.

Study	Population	Intervention	Outcome
Author, Year/Country	Number of Patients Evaluated	Type of Implant Surface	Main Complications
Maijers et al., 2013/Netherlands [23]	*n* = 80 patients	Not specified	Capsular contracture: *n* = 40 cases (50%); Local skin disorder: *n* = 3 cases (4%); Breast pain: *n* = 41 cases (51%); Infection: *n* = 5 cases (6%); Lymphadenopathy: *n* = 28 cases (35%); Changed size, form or consistence: *n* = 20 cases (25%); Loss of sensation: *n* = 9 cases (11%); Implant rotation: *n* = 1 case (1%)
Yildirimer et al., 2013/United Kingdom [25]	*n* = 10 patients	Textured implants (*n* = not specified)	Implant rupture: *n* = not specified; Capsular contracture: *n* = not specified; Discomfort and pain: *n* = not specified; Nipple discharge: *n* = not specified; Implant displacement: *n* = not specified
Brunnert, 2015/Germany [26]	*n* = 90 patients	Textured implants (*n* = 152)	Folds or wrinkles: *n* = 2 cases; Fistula: *n* = 1 case; Seroma: *n* = 2 cases
De Lorenzi et al., 2015/Italy [28]	*n* = 578 patients	Textured implants (*n* = 658)	Asymmetry: *n* = 69 cases (36.3%); Necrosis: *n* = 12 cases (6.3%); Capsular contracture: *n* = 56 cases (29.5%); Implant rupture: *n* = 22 cases (11.6%); Infection: *n* = 7 cases (3.7%); Malposition: *n* = 7 cases (3.7%)
Hammond and Schmitt, 2016/USA [29]	*n* = 79 patients	Textured implants (*n* = 134)	BIA-ALCL: *n* = 1 case (0.75%); Capsular contracture: *n* = 69 cases (51.5%); Seroma: *n* = 7 cases (5.2%); Infection: *n* = 11 cases (8.2%); Hematoma: *n* = 6 cases (4.5%); Tissue necrosis: *n* = 4 cases (3.0%); Wrinkling: *n* = 4 cases (3.0%)
Huemer et al., 2018 Austria/Germany [27]	*n* = 100 patients	Textured implants (*n* = not specified)	Capsular contracture: *n* = 1 case; Implant hypermobility: *n* = 2 cases; Implant dislocation: *n* = 2 cases; Malposition: *n* = 4 cases; Implant rupture: *n* = 1 case; Excessive size: *n* = 1 case
Hong et al., 2021/South Korea [30]	*n* = 1.314 patients	Textured implants (*n* = 2.628)	Asymmetry: *n* = 9 cases (1.03%); Capsular contracture: *n* = 18 cases (2.10%); Hematoma: *n* = 18 cases (2.10%); Infection: *n* = 6 cases (0.70%); Dissatisfaction with shape: *n* = 17 cases (1.95%); Dissatisfaction with size: *n* = 16 cases (1.83%); Waving: *n* = 3 cases (0.34%); Early seroma: *n* = 24 cases (2.70%)
Miseré and Van Der Hulst, 2022/Netherlands [24]	*n* = 197 patients	Not specified (*n* = 303)	Unsatisfactory cosmetic outcome or asymmetry: *n* = not specified (9.1%); Breast implant exposure due to infection or wound dehiscence: *n* = not specified (8.1%); Breast cancer or prophylactic breast surgery: *n* = not specified (8.1%); Seeking autologous breast reconstruction without specific reported cause: *n* = not specified (4.6%); Major concern about safety of silicone exposure: *n* = not specified (1.0%); Systemic symptoms: *n* = not specified (11.2%); Pain: *n* = not specified (13.2%); Infection: *n* = not specified (13.2%); Implant rupture: *n* = not specified (14.2%); Other reasons: *n* = not specified (2.5%); Severe capsular contracture: *n* = not specified (14.7%)

**Table 3:** Overview of descriptive characteristics of the clinical trial protocols included in the review.

Clinical Trial Protocol	Population	Intervention	Outcome
**Identification Number (NCT), Title, Year**	Patients Evaluated	Implant Type/Brand	Primary and Secondary Outcome Measures
NCT01639053 U.S. Postapproval Study of Sientra Silicone Gel Breast Implants (2012) [31]	Women receiving Sientra silicone gel breast implants for augmentation, revision augmentation, reconstruction, and revision	Sientra silicone gel breast implants	Long-term (10-year) safety of Sientra silicone gel breast implants in women.
NCT03356132 Postmarket Monitoring and Control of Safety and Efficacy of Silimed^®^ Breast Implants With Textured Surface and Polyurethane Foam (2018) [32]	Women aged 18 years or older who underwent breast augmentation using Silimed^®^ silicone gel-filled breast implants with a textured surface or Silimed^®^ silicone gel-filled breast implants with a polyurethane foam-coated surface	Silimed^®^ textured silicone gel-filled breast implant and Silimed^®^ polyurethane foam-coated silicone gel-filled breast implant	Primary outcomes: Estimation of the rates of expected and unexpected adverse events, both in the short and long term, associated with Silimed^®^ silicone breast implants with a textured surface and with a polyurethane foam-coated surface, assessed every three years over a 10-year period. Secondary outcomes: Aesthetic and general patient satisfaction, measured by Likert scales and questionnaires such as the BEQ-Brazil, the evaluator’s satisfaction with the aesthetic result, and the patient’s quality of life, assessed by the Rosemberg Global Self-Esteem Scale.
NCT03386682 Evaluation of the Safety and Performance of SYMATESE AESTHETICS ESTYME^®^ MATRIX Round and Anatomical Silicone Gel-Filled Breast Implants in the Breast Augmentation and Reconstruction—EMMIE Study (2018) [35]	Participants who met the requirements for breast augmentation or reconstruction surgery and were implanted with one or two ESTYME^®^ MATRIX breast implants	ESTYME^®^ MATRIX breast implants	Primary outcomes: Assessment of local complications and adverse events three months after implantation, such as infection, hematoma, wound healing complications, fluid accumulation, early capsular contracture, rupture and extrusion, as well as patient and surgeon satisfaction with the success of the procedure. Secondary outcomes: Assessment at one and two years, focusing on the incidence of serious adverse events and overall patient and surgeon satisfaction with the usability of the device and postprocedure outcomes.
NCT05345821 Postmarket Monitoring and Control of Safety and Efficacy of Silimed^®^ Breast Implants With Smooth Surface (STEPS S) (2022) [33]	Female births with indication for primary and secondary augmentation (review)	Silimed^®^ smooth surface breast implant	Primary outcomes: assessment of the rates of expected and unexpected adverse events, both in the short and long term, for Silimed^®^ breast implants with a smooth surface, with assessments every three years over a 10-year period. Secondary outcomes: patient satisfaction with the aesthetic result, measured by a Likert scale; overall patient satisfaction, assessed by the BEQ-Brazil questionnaire; evaluator satisfaction with the aesthetic result, also measured by a Likert scale; and patient quality of life, measured by the Rosenberg Global Self-Esteem Scale, with assessments every three years over the 10 years of the study.
NCT05336526 Evaluation of the Safety and Performance of SYMATESE AESTHETICS ESTYME^®^ MATRIX Round Microtextured Silicone Gel-Filled Breast Implants in the Breast Augmentation, Primary Intention (EMMA Study) (2022) [34]	Participants who met the requirements for bilateral breast augmentation in primary intent and were implanted with silicone gel-filled round breast implants microtexturizado ESTYME^®^ MATRIX	ESTYME^®^ MATRIX gel microtextured silicone round breast implants	Primary outcomes: Safety, assessed by the incidence of implant- or procedure-related adverse events/complications at 3 months postprocedure. Secondary outcomes: Safety assessment by the incidence of all adverse events (AEs) and serious adverse events (SAEs) at 1 and 2 years. Performance will be measured by several factors, including surgeon satisfaction with procedure duration, incision size, correct implant placement, increase in bra size, change in chest circumference, and overall patient and surgeon satisfaction at 3 months, and at 1 and 2 years.
NCT06274736 Postmarketing Cohort Study to Confirm the Safety and Performance of Motiva^®^ Sizers in Breast Augmentation and Reconstruction Procedures (2023) [36]	Women over 18 years of age who will undergo breast augmentation or reconstruction, divided into two groups: 165 participants exposed to Motiva^®^ Sizer (150 for augmentation and 15 for reconstruction) and 165 participants not exposed to Motiva^®^ Sizer (150 for augmentation and 15 for reconstruction)	Motiva^®^ Sizer Breast Implants	Primary outcomes: Incidence of complications and satisfaction of participants and surgeons, comparing the groups that used or did not use sizers, with evaluation over 3 years. Satisfaction will be measured on a 5-point Likert scale, and complications will be monitored over this period. Secondary outcome: rate of reoperations between the two groups over the same 3 years.
NCT06013514 A Prospective, Multicenter, Observational, Non Comparative, Postmarketing Surveillance Study to Obtain Clinical Outcome Data on the Nagor PERLE Range of Silicone Breast Implants When Used in Breast Implantation (2023) [37]	Women aged 18 to 65 who require breast surgery for augmentation, reconstruction or revision	PERLE Sterile Smooth Opaque Gel-Filled Breast Implants	Primary outcomes: rate of capsular contracture (Baker grades III–IV) and rate of implant rupture, assessed 10 years after surgery. Secondary outcomes: Rate of secondary surgeries required to correct complications, rate and frequency of local complications such as mild capsular contracture, hematoma, rupture, seroma, persistent pain, infection, implant displacement or extrusion, and other device- or procedure-related complications. In addition, patient satisfaction using the BREAST-Q method will be assessed, as well as the rate of any adverse events over 10 years.

**Table 4:** Number of occurrences of adverse events related to breast implants—Brazil, 2007–2023.

Adverse Event (WHO-ART)	Total	(%)
Breast augmentation	7	1.1%
Increased tissue fluids	8	1.3%
Breast cancer	7	1.1%
Breast surgery	6	1.0%
Implant-related complication	83	13.3%
Wound dehiscence	4	0.6%
Breast discomfort	5	0.8%
Pain	77	12.3%
Edema	10	1.6%
Breast hardening	9	1.4%
Fatigue	3	0.5%
Implant failure	9	1.4%
Tissue swelling	38	6.1%
Infection	38	6.1%
Breast inflammation	5	0.8%
Surgical intervention	3	0.5%
T-cell lymphoma	52	8.3%
Non-Hodgkin’s lymphoma	53	8.5%
Lymph node enlargement	3	0.5%
Mastalgia	3	0.5%
Skin nodule	3	0.5%
Reaction at the insertion site of a medical device	3	0.5%
Rupture of a medical device	12	1.9%
Burning sensation	4	0.6%
Immune system disorder	16	2.6%
Other	92	14.7%
Not reported	72	11.5%
Total	625	100.0%

Source: ANVISA. Notivisa. The data were updated on 10 September 2024 and are subject to review.

## Data Availability

The original contributions presented in this study are included in the article. Further inquiries can be directed to the corresponding authors.

## Supplementary Materials

The following supporting information can be downloaded at: https://www.mdpi.com/article/10.3390/jcm14124164/s1, Table S1: search sequence.
