# Supplementary material for: Safety and Performance of Postmarketing Breast Implants: An Integrated Review with Technovigilance Data
Source: J Clin Med. 2025 Jun 12;14(12):4164. doi: 10.3390/jcm14124164 (PMC12194552; doi:10.3390/jcm14124164)
Supplement: Supplementary file 1 [file jcm-14-04164-s001.zip › jcm-3611221-supplementary.pdf]

Data base	String	Results	Search date
BVS	(mh:"Implante Mamário" OR (Implante Mamário) OR (Breast Implantation) OR (Implantación de Mama) OR (Implantation de prothèse mammaire) OR (Implantação Mamária) OR (Implantação de Mama) OR (Implantação de Prótese Mamária) OR (Implantação de Prótese de Mama) OR (Implante de Mama) OR (Implante de Prótese Mamária) OR (Implante de Prótese de Mama) OR mh:E02.218.565.210$ OR mh:E04.617.500.210$ OR mh:E04.650.210$ OR mh:"Implantes de Mama" OR (Implantes de Mama) OR (Breast Implants) OR (Implants mammaires) OR (Prótese Interna de Mama) OR mh:E07.695.140$) AND (mh:"Silicones" OR (Silicones) OR (Siliconas) OR (Silicone) OR mh:D02.756.650.700$ OR mh:D05.750.900.850$ OR mh:D25.720.900.850$ OR mh:J01.637.051.720.900.850$ OR mh:"Géis de Silicone" OR (Géis de Silicone) OR (Silicone Gels) OR (Geles de Silicona) OR (Gels de silicone) OR mh:D02.756.650.700.755$ OR mh:D05.750.900.850.905$ OR mh:D25.720.900.850.905$ OR mh:J01.637.051.720.900.850.905$ OR mh:"Elastômeros de Silicone" OR (Elastômeros de Silicone) OR (Silicone Elastomers) OR (Elastómeros de Silicona) OR (Siloxane élastomère) OR (Borracha de Silicone) OR mh: D05.750.900.850.900$ OR mh:D25.720.327.900$ OR mh:D25.720.900.850.900$ OR mh:J01.637.051.720.327.900$ OR mh:J01.637.051.720.900.850.900$ OR mh:J01.637.412.900$ OR (Implantes de gel de silicone) OR (Silicone gel implants) OR (implantes de gel de silicone) OR (implants en gel de silicone) OR (Implantes de poliuretano) OR (Polyurethane implants) OR (Implants en polyuréthane) OR (Implantes de superfície lisa) OR (Implants à surface lisse) OR (Smooth surface implants) OR (Textured surface implants) OR (Implants à surface texturée) OR (Implantes de superficie texturizada) OR (Textured surface breast implants) OR (Implants mammaires à surface texturée) OR (Implantes mamarios con superficie texturizada) OR (Implantes mamários de superfície texturizada) OR (implantes mamários de gel de silicone) OR (implantes mamários de gel de silicone) OR (Superfícies de implantes mamários) OR (silicone gel breast implants) OR (silicone gel breast implants) OR (Breast implant surfaces) OR (implantes mamarios de gel de silicone) OR (implantes mamarios de gel de silicone) OR (Superficies de implantes mamários) OR (implants mammaires en gel de silicone) OR (implants mammaires en gel de silicone) OR (Surfaces des implants mammaires)) AND (mh:"Segurança" OR (Segurança) OR (Safety) OR (Seguridad) OR (Sécurité) OR mh:N06.850.135.060.075$ OR mh:VS4.002.001.001.007$) AND (mh:"Desempenho" OR (Desempenho) OR (Performance) OR (Actuación) OR mh:F02.784.692.351$ OR mh:N04.452.209$)	15	05.09.2024
	(mh:"Vigilância de Produtos Comercializados" OR (Product Surveillance, Postmarketing) OR (Vigilancia de Productos Comercializados) OR (Surveillance post-commercialisation des produits de santé) OR (Estudos de Avaliação Pós-Comercialização) OR (Vigilância Pós-Comercialização de Produtos para a Saúde) OR (Vigilância de Drogas Comercializadas) OR (Vigilância de Produtos para a Saúde) OR (Tecnovigilância) OR (Technovigilance) OR (Tecnovigilancia) OR mh:E05.337.800$ OR mh:VS1.001.003.001.001.005.001$) AND (mh:"Desempenho" OR (Desempenho) OR (Performance) OR (Actuación) OR mh:F02.784.692.351$ OR mh:N04.452.209$) AND (mh:"Segurança" OR (Segurança) OR (Safety) OR (Seguridad) OR (Sécurité) OR mh:N06.850.135.060.075$ OR mh:VS4.002.001.001.007$) AND (mh:"Adulto" OR (Adulto) OR (Adult) OR (Adulte) OR (Adultos) OR mh: M01.060.116$) AND (mh:"Silicones" OR (Silicones) OR (Siliconas) OR (Silicone) OR mh:D02.756.650.700$ OR mh:D05.750.900.850$ OR mh:D25.720.900.850$ OR mh:J01.637.051.720.900.850$ OR mh:"Géis de Silicone" OR (Géis de Silicone) OR (Silicone Gels) OR (Geles de Silicona) OR (Gels de silicone) OR mh:D02.756.650.700.755$ OR mh:D05.750.900.850.905$ OR mh:D25.720.900.850.905$ OR mh:J01.637.051.720.900.850.905$ OR mh:"Elastômeros de Silicone" OR (Elastômeros de Silicone) OR (Silicone Elastomers) OR (Elastómeros de Silicona) OR (Siloxane élastomère) OR (Borracha de Silicone) OR mh: D05.750.900.850.900$ OR mh:D25.720.327.900$ OR mh:D25.720.900.850.900$ OR mh:J01.637.051.720.327.900$ OR mh:J01.637.051.720.900.850.900$ OR mh:J01.637.412.900$ OR (Implantes de gel de silicone) OR (Silicone gel implants) OR (implantes de gel de silicone) OR (implants en gel de silicone) OR (Implantes de poliuretano) OR (Polyurethane implants) OR (Implants en polyuréthane) OR (Implantes de superfície lisa) OR (Implants à surface lisse) OR (Smooth surface implants) OR (Textured surface implants) OR (Implants à surface texturée) OR (Implantes de superficie texturizada) OR (Textured surface breast implants) OR (Implants mammaires à surface texturée) OR (Implantes mamarios con superficie texturizada) OR (Implantes mamários de superfície texturizada) OR (implantes mamários de gel de silicone) OR (implantes mamários de gel de silicone) OR (Superfícies de implantes mamários) OR (silicone gel breast implants) OR (silicone gel breast implants) OR (Breast implant surfaces) OR (implantes mamarios de gel de silicone) OR (implantes mamarios de gel de silicone) OR (Superficies de implantes mamários) OR (implants mammaires en gel de silicone) OR (implants mammaires en gel de silicone) OR (Surfaces des implants mammaires)) AND (mh:"Implante Mamário" OR (Implante Mamário) OR (Breast Implantation) OR (Implantación de Mama) OR (Implantation de prothèse mammaire) OR (Implantação Mamária) OR (Implantação de Mama) OR (Implantação de Prótese Mamária) OR (Implantação de Prótese	1	

	de Mama) OR (Implante de Mama) OR (Implante de Prótese Mamária) OR (Implante de Prótese de Mama) OR mh:E02.218.565.210$ OR mh:E04.617.500.210$ OR mh:E04.650.210$ OR mh:"Implantes de Mama" OR (Implantes de Mama) OR (Breast Implants) OR (Implants mammaires) OR (Prótese Interna de Mama) OR mh:E07.695.140$)		
Pubmed	**#1** "Breast Implantation"[Mesh] OR (Breast Implantations) OR (Implantation, Breast) OR (Implantations, Breast) OR (Breast Prosthesis Implantation) OR (Breast Prosthesis Implantations) OR (Implantation, Breast Prosthesis) OR (Implantations, Breast Prosthesis) OR (Prosthesis Implantation, Breast) OR (Prosthesis Implantations, Breast)<br>**#2** "Breast Implants"[Mesh] OR (Implants, Breast) OR (Breast Implant) OR (Implant, Breast) OR (Breast Prosthesis, Internal) OR (Breast Prostheses, Internal) OR (Internal Breast Prostheses) OR (Internal Breast Prosthesis) OR (Prostheses, Internal Breast) OR (Prosthesis, Internal Breast)<br>**#1 OR #2 = #3**<br>**#4** "Safety"[Mesh] OR (Safeties)<br>**#5** "Patient Safety"[Mesh] OR (Patient Safeties) OR (Safeties, Patient) OR (Safety, Patient)<br>**#6** "Safety-Based Medical Device Withdrawals"[Mesh] OR (Safety Based Medical Device Withdrawals) OR (Medical Device Safety Withdrawals) OR (Biomedical Device Safety Withdrawals) OR (Safety-Based Biomedical Device Withdrawals) OR (Safety Based Biomedical Device Withdrawals)<br>**#7** "Equipment Safety"[Mesh] OR (Safety, Equipment) OR (Hazards, Equipment) OR (Equipment Hazard) OR (Equipment Hazards) OR (Hazard, Equipment) OR (Medical Device Safety) OR (Device Safety, Medical) OR (Safety, Medical Device) OR (Device Safety) OR (Safety, Device)<br>**#4 OR #5 OR #6 OR #7 = #8**<br>**#9** Performance<br>**#10** "Silicone Gels"[Mesh] OR (Gels, Silicone) OR "Silicones"[Mesh] OR (Silicone) OR "Silicone Elastomers"[Mesh] OR (Rubber Silicone) OR (Elastomers, Silicone) OR (Silicone Elastomer) OR (Silicone Rubber) OR (Elastosil) OR (Elastosils) OR (Microfil) OR (Microfils) OR (SE-30) OR (SE30) OR (SE 30) OR (Silicone gel implants) OR (Polyurethane implants) OR (Smooth surface implants) OR (Textured surface implants) OR (Textured surface breast implants) OR (Silicone gel breast implants) OR (Breast implant surfaces)<br>**#3 AND #8 AND #9 AND #10**	66	05.09.2024
	**#1** "Breast Implantation"[Mesh] OR (Breast Implantations) OR (Implantation, Breast) OR (Implantations, Breast) OR (Breast Prosthesis Implantation) OR (Breast Prosthesis Implantations) OR (Implantation, Breast Prosthesis) OR (Implantations, Breast Prosthesis) OR (Prosthesis Implantation, Breast) OR (Prosthesis Implantations, Breast)<br>**#2** "Breast Implants"[Mesh] OR (Implants, Breast) OR (Breast Implant) OR (Implant, Breast) OR (Breast Prosthesis, Internal) OR (Breast Prostheses, Internal) OR (Internal Breast Prostheses) OR (Internal Breast Prosthesis) OR (Prostheses, Internal Breast) OR (Prosthesis, Internal Breast)<br>**#1 OR #2 = #3**<br>**#4** "Safety"[Mesh] OR (Safeties)<br>**#5** "Patient Safety"[Mesh] OR (Patient Safeties) OR (Safeties, Patient) OR (Safety, Patient)<br>**#6** "Safety-Based Medical Device Withdrawals"[Mesh] OR (Safety Based Medical Device Withdrawals) OR (Medical Device Safety Withdrawals) OR (Biomedical Device Safety Withdrawals) OR (Safety-Based Biomedical Device Withdrawals) OR (Safety Based Biomedical Device Withdrawals)<br>**#7** "Equipment Safety"[Mesh] OR (Safety, Equipment) OR (Hazards, Equipment) OR (Equipment Hazard) OR (Equipment Hazards) OR (Hazard, Equipment) OR (Medical Device Safety) OR (Device Safety, Medical) OR (Safety, Medical Device) OR (Device Safety) OR (Safety, Device)<br>**#4 OR #5 OR #6 OR #7 = #8**<br>**#9** Performance<br>**# 10** "Product Surveillance, Postmarketing"[Mesh] OR (Evaluation Studies, Postmarketing) OR (Evaluation Study, Postmarketing) OR (Postmarketing Evaluation Studies) OR (Postmarketing Evaluation Study) OR (Studies, Postmarketing Evaluation) OR (Study, Postmarketing Evaluation) OR (Postmarketing Product Surveillance) OR (Postmarketing Product Surveillances) OR (Product Surveillances, Postmarketing) OR (Surveillance, Postmarketing Product) OR (Surveillances, Postmarketing Product) OR (Product Surveillance, Post-	2	

	Marketing) OR (Post-Marketing Product Surveillance) OR (Post-Marketing Product Surveillances) OR (Product Surveillance, Post Marketing) OR (Product Surveillances, Post-Marketing) OR (Surveillance, Post-Marketing Product) OR (Surveillances, Post-Marketing Product) OR (Drug Surveillance, Postmarketing) OR (Drug Surveillances, Postmarketing) OR (Postmarketing Drug Surveillance) OR (Postmarketing Drug Surveillances) OR (Surveillance, Postmarketing Drug) OR (Surveillances, Postmarketing Drug)<br>**#11** "Silicone Gels"[Mesh] OR (Gels, Silicone) OR "Silicones"[Mesh] OR (Silicone) OR "Silicone Elastomers"[Mesh] OR (Rubber Silicone) OR (Elastomers, Silicone) OR (Silicone Elastomer) OR (Silicone Rubber) OR (Elastosil) OR (Elastosils) OR (Microfil) OR (Microfils) OR (SE-30) OR (SE30) OR (SE 30) OR (Silicone gel implants) OR (Polyurethane implants) OR (Smooth surface implants) OR (Textured surface implants) OR (Textured surface breast implants) OR (Silicone gel breast implants) OR (Breast implant surfaces)<br>**#3 AND #8 AND #9 AND #10 AND #11**		
Embase	('breast augmentation'/exp OR 'augmentation mammoplasty' OR 'augmentation, breast' OR 'breast enlargement' OR 'breast implantation' OR 'enlargement, breast' OR 'mammoplasty, augmentation' OR 'breast augmentation' OR 'breast endoprosthesis'/exp OR 'breast implant' OR 'breast implants' OR 'breast prostheses, internal' OR 'cpg (breast endoprosthesis)' OR 'endoprosthesis, breast' OR 'implant, breast' OR 'internal breast prostheses' OR 'internal breast prosthesis' OR 'mamma endoprosthesis' OR 'mammary endoprosthesis' OR 'memoryshape' OR 'mentor (breast endoprosthesis)' OR 'mentor memorygel' OR 'mentor memorygel xtra' OR 'mentor spectrum' OR 'natrelle' OR 'sientra' OR 'silicone gel/saline-filled breast implant' OR 'breast endoprosthesis') AND ('safety'/exp OR 'safety management' OR 'safety precaution' OR 'safety protection' OR 'safety regulation' OR 'safety' OR 'patient safety'/exp OR 'patient safety' OR 'device recall'/exp OR 'medical device recall' OR 'medical device recalls' OR 'safety-based medical device withdrawals' OR 'device recall' OR 'device safety'/exp OR 'equipment safety' OR 'device safety') AND ('performance'/exp OR 'performance test' OR 'progressive ratio performance' OR 'performance') AND ('silicone gel'/exp OR 'silicone gels' OR 'silicone gel' OR 'silicone derivative'/exp OR 'silicones' OR 'silicone derivative' OR 'silastic'/exp OR 'silastic elastomer' OR 'silicon rubber' OR 'silicone elastomer' OR 'silicone elastomere' OR 'silicone elastomers' OR 'silicone rubber' OR 'silastic' OR 'polyurethan'/exp OR 'smooth surface'/exp OR 'textured breast implant'/exp)	11	05.09.2024
	('breast augmentation'/exp OR 'augmentation mammoplasty' OR 'augmentation, breast' OR 'breast enlargement' OR 'breast implantation' OR 'enlargement, breast' OR 'mammoplasty, augmentation' OR 'breast augmentation' OR 'breast endoprosthesis'/exp OR 'breast implant' OR 'breast implants' OR 'breast prostheses, internal' OR 'cpg (breast endoprosthesis)' OR 'endoprosthesis, breast' OR 'implant, breast' OR 'internal breast prostheses' OR 'internal breast prosthesis' OR 'mamma endoprosthesis' OR 'mammary endoprosthesis' OR 'memoryshape' OR 'mentor (breast endoprosthesis)' OR 'mentor memorygel' OR 'mentor memorygel xtra' OR 'mentor spectrum' OR 'natrelle' OR 'sientra' OR 'silicone gel/saline-filled breast implant' OR 'breast endoprosthesis') AND ('safety'/exp OR 'safety management' OR 'safety precaution' OR 'safety protection' OR 'safety regulation' OR 'safety' OR 'patient safety'/exp OR 'patient safety' OR 'device recall'/exp OR 'medical device recall' OR 'medical device recalls' OR 'safety-based medical device withdrawals' OR 'device recall' OR 'device safety'/exp OR 'equipment safety' OR 'device safety') AND ('performance'/exp OR 'performance test' OR 'progressive ratio performance' OR 'performance') AND ('silicone gel'/exp OR 'silicone gels' OR 'silicone gel' OR 'silicone derivative'/exp OR 'silicones' OR 'silicone derivative' OR 'silastic'/exp OR 'silastic elastomer' OR 'silicon rubber' OR 'silicone elastomer' OR 'silicone elastomere' OR 'silicone elastomers' OR 'silicone rubber' OR 'silastic' OR 'polyurethan'/exp OR 'smooth surface'/exp OR 'textured breast implant'/exp) AND ('postmarketing surveillance'/exp OR 'post-market evaluation study' OR 'post-market surveillance' OR 'post-marketing product surveillance' OR 'post-marketing surveillance' OR 'postmarket surveillance' OR 'postmarketing evaluation study' OR 'postmarketing product surveillance' OR 'product surveillance program' OR 'product surveillance, postmarketing' OR 'surveillance, postmarketing' OR 'postmarketing surveillance')	3	
Clinical Trials	Safety and performance AND Breast Implants	13	05.09.2024
	Post-market Surveillance AND Breast Implants	2	
